# Development and validation of a pediatric spine surgical invasiveness index

**DOI:** 10.1007/s43390-025-01106-y

**Published:** 2025-05-13

**Authors:** Vivien Chan, Adeesya Gausper, Andrew Chan-Tai-Kong, Andy M. Liu, Suhas Etigunta, Justin K. Scheer, Lindsay M. Andras, David L. Skaggs

**Affiliations:** 1 Spine Center, Cedars-Sinai Medical Center, 444 S San Vicente Blvd, Ste 900, Los Angeles, CA 90048 USA; 2 Division of Neurosurgery, University of Alberta Hospital, Edmonton, Alberta Canada; 3 Jackie and Gene Autry Orthopedic Center, Children’s Hospital Los Angeles, Los Angeles, CA USA

**Keywords:** Pediatric, Scoliosis, Risk prognostication, Risk stratification, Morbidity, Mortality

## Abstract

**Purpose:**

Surgical invasiveness indices have been used in adult spine surgery to characterize the invasiveness of complex procedures and for risk stratification. This has not been studied in the pediatric population. The purpose of this study was to develop and validate a surgical invasiveness index for pediatric spinal deformity surgery.

**Methods:**

The National Surgical Quality Improvement Program (NSQIP) Pediatric database was queried between the years 2016–2022. Patients were included if they were <18 years of age, received posterior or anterior-posterior spinal fusion surgery, and had a diagnosis of spinal deformity. The study cohort was divided into a derivation cohort and a validation cohort. A multivariable linear regression analysis was performed to identify surgical components associated with operative time. Surgical components of interest included number of posterior fusion levels, number of anterior fusion levels, pelvic instrumentation, posterior column osteotomies, three-column osteotomies, and prior spinal deformity surgery. Statistically significant variables were used to establish a pediatric spinal deformity surgical invasiveness index. The score was assessed and validated using linear and logistic regression analysis and receiver operating characteristic curve analysis on operative time and allogeneic transfusion.

**Results:**

There were 37,658 patients included (Derivation cohort: 26,372; Validation cohort: 11,286). In the linear regression analysis, more posterior fusion levels (7–12 levels: 0.54, p<0.001;>12 levels: 1.40, p<0.001), anterior fusion 1–3 levels (2.42, p<0.001), anterior fusion ≥4 levels (2.93, p<0.001), pelvic instrumentation (0.79, p<0.001), and previous spinal deformity surgery (0.44, p<0.001) were associated with longer operative time. Each level of posterior column osteotomy (0.13, p<0.001) and three-column osteotomy (0.61, p<0.001) were associated with increased operative time. Points were assigned to each surgical component: 7–12 posterior fusion levels (4 pts), >12 posterior fusion levels (11 pts), anterior fusion 1-3 levels (19 pts), anterior fusion ≥4 levels (23 pts), pelvic instrumentation (6 pts), previous spinal deformity surgery (3 pts), posterior column osteotomy (1 pt per level), and three-column osteotomy (5 pts per level). In the derivation cohort, each point was associated with an increase in operative time by 0.13 hours (R^2^=0.16, p<0.001). In the validation cohort, each point was associated with an increase in operative time by 0.12 hours (R^2^=0.15, p<0.001). In the derivation cohort, the area under the curve (AUC) for operative time ≥8 hours and allogeneic transfusion were 0.74 and 0.71, respectively. In the validation cohort, the AUC for operative time ≥8 hours and allogeneic transfusion were 0.74 and 0.70, respectively.

**Conclusion:**

A pediatric spinal deformity surgical invasiveness index was created and predictive of prolonged operative time and allogeneic transfusion. This is the first quantitative tool to measure the extent of surgical interventions in pediatric spine surgery.

**Supplementary Information:**

The online version contains supplementary material available at 10.1007/s43390-025-01106-y.

## Introduction

Surgical correction of pediatric spinal deformity varies in levels of fusion involved, osteotomies, and need for pelvic instrumentation. These factors contribute to surgical complexity and have implications on operative time and blood transfusion needs, which have been associated with adverse outcomes [1–5]. The heterogeneity of pediatric deformity cases contributes to a wide range of outcomes and complications, highlighting the need for a robust system to assess surgical invasiveness. In adult spine surgery, surgical invasiveness indices have been developed to quantify the complexity of procedures and guide risk stratification for adverse outcomes [6–9]. However, a validated invasiveness index specific to pediatric spinal deformity surgery has not yet been developed and outcomes of pediatric spinal surgery cannot be directly inferred from adult data [10, 11]. The ability to assess perioperative demands is critical for risk stratification, optimizing resources, and improving patient outcomes.

This study seeks to address this gap through development and validation of a surgical invasiveness index for pediatric spinal deformity surgery. We utilized the NSQIP Pediatric database to identify key surgical components that contribute to increased operative time and allogeneic transfusion requirements. The proposed index aims to serve as a valuable tool for preoperative risk stratification to inform clinical decision-making and patient counseling in pediatric spinal deformity surgery.

## Methods

### Study design and data source

The American College of Surgeons National Surgical Quality Improvement Program (NSQIP) Pediatric database for years 2016–2022 was used for this study. NSQIP is a nationally validated, risk-adjusted, outcomes-based program that measures the quality of surgical care. In each participating hospital, a trained Surgical Clinical Reviewer collects preoperative through 30-day post-operative data on randomly selected patients. Data are entered online in a secure, web-based platform available for open access by request.

Patients were included in this study if they were (1) <18 years of age, (2) received posterior or anterior-posterior spinal fusion surgery, and 3) had a diagnosis of spinal deformity. Posterior or anterior-posterior spinal fusion for spinal deformity were isolated by CPT codes 22800, 22802, or 22804. Spinal deformities included idiopathic scoliosis, neuromuscular scoliosis, syndromic scoliosis, congenital spinal deformities, spondylolisthesis, and kyphosis. Patients with anterior only spinal fusion were excluded. The NSQIP Pediatric database collection excludes: (1) patients 18 years of age or older, (2) cases involving hyperthermic intraperitoneal chemotherapy, (3) ASA score of 6, (4) concurrent case by different surgical team under the same anesthetic, (5) multiple cases within 30 days, (6) transplant cases, and (7) trauma and abuse cases.

Patient demographics and surgical metrics were extracted from the database. The primary CPT code and concurrent CPT codes were used to characterize number of posterior fusion levels (<7, 7–12, >12 surgical levels), number of anterior fusion levels (1–3, ≥4 surgical levels), addition of pelvic instrumentation, addition of posterior column osteotomies, and addition of three-column osteotomies (Supplementary Table 1).

### Development and validation of the index

**Fig. 1:**
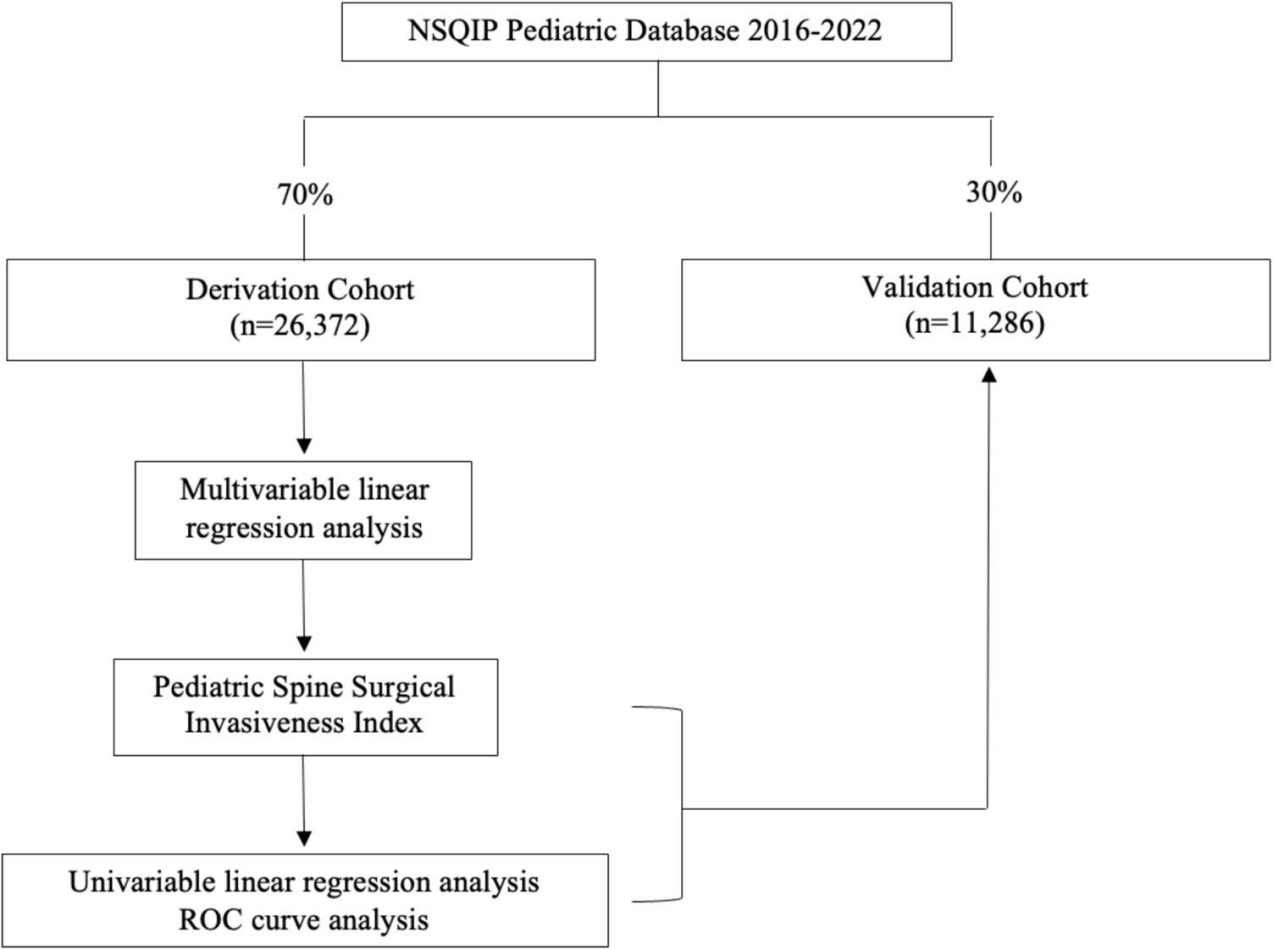
Flowchart of study

The study cohort was divided into a derivation cohort and a validation cohort using a 70:30 random split (Fig. 1). A multivariable linear regression analysis with operative time in hours was performed to identify surgical components associated with operative time for the derivation cohort. Variables in the model included number of posterior spinal fusion levels (<7, 7–12, >12 surgical levels), number of anterior spinal fusion levels (1–3, ≥4 surgical levels), posterior column osteotomy, three-column osteotomy, pelvic instrumentation, and previous spinal deformity surgery. The B values from the regression analysis were used to assign points to each surgical component for the pediatric spine surgical invasiveness index.

The total score for the pediatric spine surgical invasiveness index was determined for each patient. Univariable linear regression analysis was performed to determine the association between the score and operative time (in hours) in both the derivation and validation cohort. Univariable logistic regression analysis was performed to determine the association between the score and allogeneic transfusion in the derivation and validation cohort. The adjusted R^2^ values were determined. Operative time was dichotomized to (1) <8 hours and (2) ≥8 hours. The pediatric spine surgical invasiveness index was validated using receiver operating characteristic (ROC) curve analysis. The area under the curve (AUC) was determined for prolonged operative time (≥8 hours) and allogeneic transfusion for both the derivation cohort and validation cohort. Kolmogorov-Smirnov (K-S) statistic was reported for each ROC curve analysis. The mean score was reported for the dichotomous outcomes for the derivation and validation cohort.

### Multivariable logistic regression analysis using the pediatric spine surgical invasiveness index

Multivariable logistic regression analysis was performed to determine the association between the pediatric spine surgical invasiveness index score and (1) prolonged operative time (>8 hours) and (2) allogeneic transfusion. The models were adjusted for age (years), gender, ASA score, body mass index (BMI), etiology of scoliosis (idiopathic, congenital, kyphosis, neuromuscular, syndromic), and use of cell salvage. Variables were chosen a priori based on clinical relevance and previous literature.

All statistical analysis was performed using IBM SPSS Statistics version 29.0.2.0. Alpha value for significance was 0.05, which was set a priori. Listwise deletion was used for missing data for complete case analysis.

## Results

### Patient and surgical characteristics

There were 26,372 patients in the derivation cohort (Table 1). The mean age was 13.8±2.6 years. Of the 26,372, 68.1% (n=17,949) were female. A majority of the patients had idiopathic (64.4%, n= 16,982) or neuromuscular (20.1%, n=5298) spinal deformities. Of the 26,372 patients, 11.0% (n=2900) had <7 posterior fusion levels, 51.3% (n=13,526) had 7–12 posterior fusion levels, and 37.7% (n=9946) had >12 posterior fusion levels. Anterior fusion of 1–3 levels was performed in 0.7% (n=181) and anterior fusion of ≥4 levels was performed in 0.6% (n=155). Pelvic instrumentation was used in 8.8% (n=2314). Posterior column osteotomies were performed in 33.3% (n=8778). Three-column osteotomies were performed in 1.1% (n=279). In the derivation cohort, 9.5% (n= 2500) had previous spinal deformity surgery. The mean operative time was 4.8±1.8 hours. In the derivation cohort, patients who received <7 posterior fusion levels without anterior fusion, pelvic instrumentation, osteotomies, or previous spinal surgery, the mean operative time was 3.8±1.5 hours.

**Table 1:** Patient and surgical characteristics

	Derivation cohort (n=26,372)	Validation cohort (n=11,286)
**Age (years)**	13.8± 2.6	13.9± 2.7
**Female**	17,949 (68.1%)	7687 (68.1%)
**Etiology**		
Congenital	1783 (6.8%)	722 (6.4%)
Idiopathic	16,982 (64.4%)	7353 (65.2%)
Kyphosis	1255 (4.8%)	493 (4.4%)
Neuromuscular	5298 (20.1%)	2261 (20.0%)
Syndromic	1054 (4.0%)	457 (4.0%)
Posterior fusion levels		
<7 levels	2900 (11.0%)	1242 (11.0%)
7–12 levels	13,526 (51.3%)	5908 (52.3%)
>12 levels	9946 (37.7%)	4136 (36.6%)
Anterior fusion levels		
1–3 levels	181 (0.7%)	67 (0.6%)
≥4 levels	155 (0.6%)	50 (0.4%)
**Pelvic instrumentation**	2314 (8.8%)	999 (8.9%)
**Posterior column osteotomies**	8778 (33.3%)	3,657 (32.4%)
**Three column osteotomies**	279 (1.1%)	100 (0.9%)
**Previous spinal deformity surgery**	2500 (9.5%)	1068 (9.5%)
**Operative time (hours)**	4.8± 1.8	4.8± 1.8

There were 11,286 patients in the validation cohort (Table 1). The mean age was 13.9±2.7 years. Of the 11,286, 68.1% (n=7687) were female. A majority of the patients had idiopathic (65.2%, n= 7353) or neuromuscular (20.0%, n=2261) spinal deformities. Of the 11,286 patients, 11.0% (n=1242) had <7 posterior fusion levels, 52.3% (n=5908) had 7–12 posterior fusion levels, and 36.6% (n=4136) had >12 posterior fusion levels. Anterior fusion of 1–3 levels was performed in 0.6% (n=67) and anterior fusion of ≥4 levels was performed in 0.4% (n=50). Pelvic instrumentation was used in 8.9% (n=999). Posterior column osteotomies were performed in 32.4% (n=3657). Three-column osteotomies were performed in 0.9% (n=100). In the validation cohort, 9.5% (n=1068) had previous spinal deformity surgery. The mean operative time was 4.8±1.8 hours for the entire cohort. In the validation cohort, patients who received <7 posterior fusion levels without anterior fusion, pelvic instrumentation, osteotomies, or previous spinal surgery, the mean operative time was 3.8±1.5 hours.

### Multivariable linear regression analysis on operative time and development of index

Compared to <7 posterior fusion levels, 7–12 posterior fusion levels (0.54 [95%CI 0.47–0.61], p<0.001) and >12 posterior fusion levels (1.40 [95%CI 1.33–1.47], p<0.001) was associated with longer operative time. Anterior fusion 1–3 levels (2.42 [95%CI 2.18–2.66], p<0.001) and anterior fusion ≥4 levels (2.93 [95%CI 2.67–3.19], p<0.001) were associated with longer operative time. Addition of pelvic instrumentation was associated with an increase in operative time by 0.79 hours (0.79 [95%CI 0.71–0.86], p<0.001). Each level of posterior column osteotomy was associated with an increase in operative time by 0.13 hours (0.13 [95%CI 0.11–0.14], p<0.001). Three-column osteotomies were associated with an increase in operative time by 0.61 hours per level (0.61 [95%CI 0.47–0.76], p<0.001). Previous spinal deformity surgery was associated with an increase in operative time by 0.44 hours (0.44 [95%CI 0.37–0.51], p<0.001). The correlation coefficient for the model was 0.40 and the adjusted R^2^ value was 0.16 (Table 2).

**Table 2:** Multivariable linear regression analysis on surgical components and operative time (hours)

	B	95% CI	p value
**Posterior fusion levels (ref. <7 levels)**			
7–12 levels	0.54	0.47–0.61	<0.001*
>12 levels	1.40	1.33–1.47	<0.001*
**Anterior fusion levels**			
1–3 levels	2.38	2.14–2.62	<0.001*
≥4 levels	2.84	2.58–3.10	<0.001*
**Pelvic instrumentation**	0.79	0.71–0.86	<0.001*
**Posterior column osteotomies (per level)**	0.13	0.11–0.14	<0.001*
**Three column osteotomies (per level)**	0.61	0.47–0.76	<0.001*
**Previous spinal deformity surgery**	0.44	0.37–0.51	<0.001*

*Signifies statistical significance, alpha level 0.05

Based on the B values from the regression model, points were assigned to each surgical component (Table 3). Compared to <7 posterior fusion levels, 7–12 fusion levels and >12 fusion levels were assigned 4 points and 11 points, respectively. Anterior fusion of 1–3 levels was assigned 19 points and anterior fusion of ≥4 levels was assigned 23 points. Pelvic instrumentation was assigned 6 points. Posterior column osteotomies were assigned 1 point per level. Three-column osteotomies were assigned 5 points per level. Previous deformity surgery was assigned 3 points. The mean score for the derivation and validation cohort were 8.2±5.7 [range 0–61] and 8.1±5.6 [range 0–46], respectively.

**Table 3:** Pediatric spine surgical invasiveness index

	Points
**Posterior fusion levels (ref. <7 levels)**	
7–12 levels	4
>12 levels	11
**Anterior fusion levels**	
1–3 levels	19
≥4 levels	23
**Pelvic instrumentation**	6
**Posterior column osteotomies (per level)**	1
**Three column osteotomies (per level)**	5
**Previous spinal deformity surgery**	3

### Pediatric spine surgical invasiveness index validation on derivation cohort

**Fig. 2:**
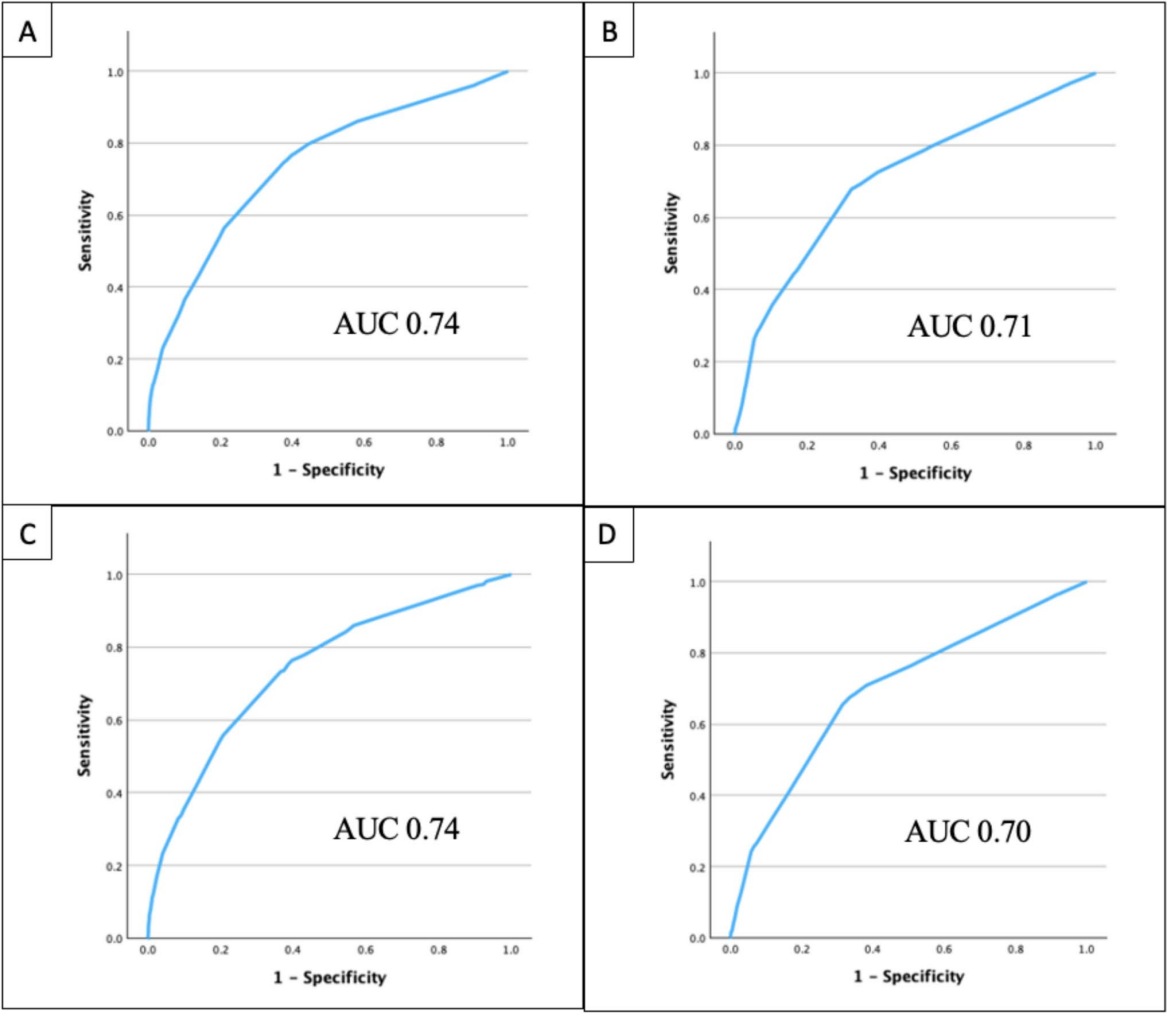
ROC curves for derivation cohort for **A** operative time>8 hours and **B** allogeneic transfusion. ROC curves for validation cohort for **C** operative time>8 hours and **D** allogeneic transfusion.

In the univariable linear regression analysis on the derivation cohort, each point of the index was associated with an increase in operative time by 0.13 hours (0.13 [95%CI 0.12–0.13], p<0.001) (Table 4). The adjusted R^2^ value was 0.16. For the derivation cohort, the AUC for operative time ≥8 hours was 0.74 and the K-S statistic was 0.37 (Fig. 2). The mean score for patients with operative time <8 hours and ≥8 hours were 7.9±5.3 and 13.8±8.2, respectively. The rates of prolonged operative time (≥8 hours) for a score less than 10, 10–30, and over 30 were 2.4%, 9.9%, and 57.1%, respectively.

**Table 4:** Univariable linear regression analysis on operative time (hours)

	B	95% CI	p value
**Derivation cohort**			
Index Score	0.13	0.12–0.13	<0.001*
**Validation cohort**			
Index Score	0.12	0.12–0.13	<0.001*

*Signifies statistical significance, alpha level 0.05

In a univariable logistic regression analysis, each point of the index was significantly associated with increased odds of allogeneic transfusion (OR 1.14 [95%CI 1.14–1.15], p<0.001) (Table 5). The adjusted R^2^ value was 0.142. The AUC for allogeneic transfusion was 0.71 and the K-S statistic was 0.36 (Fig. 2). The mean score for patients that did and did not receive allogeneic transfusion were 11.7±6.5 and 7.2±5.0, respectively. The rates of allogeneic transfusion for a score less than 10, 10–30, and over 30 were 11.0%, 34.9%, and 67.7%, respectively.

**Table 5:** Univariable logistic regression analysis on allogeneic transfusion

	OR	95% CI	p value
**Derivation cohort**			
Index score	1.14	1.14–1.15	<0.001*
**Validation cohort**			
Index score	1.13	1.12–1.14	<0.001*

*Signifies statistical significance, alpha level 0.05

### Pediatric spine surgical invasiveness index validation on validation cohort

In the validation cohort, each point of the index was associated with an increase in operative time by 0.12 hours (0.12 [95%CI 0.12–0.13], p<0.001) (Table 4). The adjusted R^2^ value was 0.15. For the validation cohort, the AUC for operative time ≥8 hours was 0.74 and the K-S statistic was 0.37 (Fig. 2). The mean score for patients with operative time <8 hours and ≥8 hours were 7.7±5.3 and 13.5±7.7, respectively. The rates of prolonged operative time (≥8 hours) for a score less than 10, 10–30, and over 30 were 2.3%, 9.5%, and 49.1%, respectively.

In a univariable logistic regression analysis, each point of the index was significantly associated with increased odds of allogeneic transfusion (OR 1.13 [95%CI 1.13–1.14], p<0.001) (Table 5). The adjusted R^2^ value was 0.127. The AUC for allogeneic transfusion was 0.70 and the K-S statistic was 0.34 (Fig. 2). The mean score for patients that did and did not receive allogeneic transfusion was 11.4±6.5 and 7.2±5.0, respectively. The rates of allogeneic transfusion for a score less than 10, 10–30, and over 30 were 11.5%, 34.5%, and 67.3%, respectively.

### Multivariable logistic regression analysis using pediatric spine surgical invasiveness score

In a multivariable logistic regression analysis, the pediatric spine surgical invasiveness index was associated with higher odds of prolonged operative time (OR 1.12 [95% CI 1.11–1.13], p<0.001) (Table 6). Age in years (OR 1.04 [95% CI 1.01–1.06], p=0.002), ASA score 3 (OR 1.71 [95% CI 1.34–2.16], p<0.001), ASA score 4 (OR 1.99 [95% CI 1.42–2.77], p<0.001), and use of intraoperative cell salvage (OR 2.04 [95% CI 1.77–2.36], p<0.001) were associated with higher odds of prolonged operative time. Compared to patients with idiopathic scoliosis, patients with congenital scoliosis (OR 2.16 [95% CI 1.74–2.68], p<0.001), kyphosis (OR 2.26 [95% CI 1.81–2.81], p<0.001), neuromuscular scoliosis (OR 1.53 [95% CI 1.28–1.82], p<0.001), and syndromic scoliosis (OR 1.78 [95% CI 1.38–2.30], p<0.001) had higher odds of prolonged operative time. Female gender (p=0.101), BMI (p=0.451), and ASA score 2 (p=0.522) were not associated with prolonged operative time.

**Table 6:** Multivariable logistic regression analysis on Pediatric Spine Surgical Invasiveness Index score and operative time >8 hours

	OR	95% CI	p value
**Age (years)**	1.04	1.01–1.06	0.002*
**Female**	0.91	0.81–1.02	0.101
**ASA (ref. ASA 1)**			<0.001*
ASA 2	1.07	0.86–1.33	0.522
ASA 3	1.71	1.34–2.16	<0.001*
ASA 4	1.99	1.42–2.77	<0.001*
** BMI**	1.00	1.00–1.00	0.451
**Etiology (ref. Idiopathic)**			<0.001*
Congenital	2.16	1.74–2.68	<0.001*
Kyphosis	2.26	1.81–2.81	<0.001*
Neuromuscular	1.53	1.28–1.82	<0.001*
Syndromic	1.78	1.38–2.30	<0.001*
** Intraoperative cell salvage**	2.04	1.77–2.36	<0.001*
** Pediatric spine surgical invasiveness index**	1.12	1.11–1.13	<0.001*

*Signifies statistical significance, alpha level 0.05

In a multivariable logistic regression analysis, the pediatric spine surgical invasiveness index was associated with higher odds of allogeneic transfusion (OR 1.09 [95% CI 1.08–1.10], p<0.001) (Table 7). Female gender (OR 1.44 [95% CI 1.34–1.56], p<0.001), ASA score 3 (OR 1.89 [95% CI 1.66–2.14], p<0.001), ASA score 4 (OR 3.44 [95% CI 2.80–4.24], p<0.001), and use of intraoperative cell salvage (OR 1.18 [95% CI 1.10–1.28], p<0.001) were associated with higher odds of allogeneic transfusion. Compared to patients with idiopathic scoliosis, patients with congenital scoliosis (OR 1.57 [95% CI 1.37–1.79], p<0.001), kyphosis (OR 1.28 [95% CI 1.09–1.50], p=0.003), neuromuscular scoliosis (OR 2.56 [95% CI 2.31–2.84], p<0.001), and syndromic scoliosis (OR 1.80 [95% CI 1.54–2.10], p<0.001) had higher odds of allogeneic transfusion. Age in years (OR 0.96 [95% CI 0.94–0.97], p<0.001) and BMI (OR 0.99 [95% CI 0.98–0.99], p<0.001) were associated with lower odds of allogeneic transfusion. ASA score 2 was not associated with odds of allogeneic transfusion (p=0.528).

**Table 7:** Multivariable logistic regression analysis on pediatric spine surgical invasiveness index score and allogeneic transfusion

	OR	95% CI	p value
**Age (years)**	0.96	0.94–0.97	<0.001*
**Female**	1.44	1.34–1.56	<0.001*
**ASA (ref. ASA 1)**			<0.001*
ASA 2	0.96	0.86–1.08	0.528
ASA 3	1.89	1.66–2.14	<0.001*
ASA 4	3.44	2.80–4.24	<0.001*
** BMI**	0.99	0.98–0.99	<0.001*
**Etiology (ref. Idiopathic)**			<0.001*
Congenital	1.57	1.37–1.79	<0.001*
Kyphosis	1.28	1.09–1.50	0.003*
Neuromuscular	2.56	2.31–2.84	<0.001*
Syndromic	1.80	1.54–2.10	<0.001*
** Intraoperative cell salvage**	1.18	1.10–1.28	<0.001*
** Pediatric spine surgical invasiveness index**	1.09	1.08–1.10	<0.001*

*Signifies statistical significance, alpha level 0.05

## Discussion

Surgical invasiveness indices have been studied in adult spine patients for various spinal pathologies. However, a surgical invasiveness index has not been established for pediatric spine surgery. We developed and validated a surgical invasiveness index using 37,658 pediatric patients who received surgery for spinal deformity. In our index we included number of posterior fusion levels, number of anterior fusion levels, pelvic instrumentation, posterior column osteotomies, three-column osteotomies, and history of previous spinal surgery. All surgical components were associated with increased operative time. Each point in the surgical invasiveness index was associated with an increase in operative time by 0.13 hours (8 minutes). The surgical invasiveness index had good discrimination for prolonged operative time (>8 hours) and allogeneic transfusion. Adjusting for patient factors and surgical factors, the pediatric spine surgical invasiveness index was an independent risk factor for increased odds of allogeneic transfusion and prolonged operative time.

The methodology for developing our surgical invasiveness index was previously described by Mirza et al. [7] Mirza et al. used a prospective cohort of 1745 adult spine patients to develop a surgical invasiveness index based on operative time [7]. The index included anterior decompression, anterior fusion, anterior instrumentation, posterior decompression, posterior fusion, and posterior instrumentation [7]. Each point on the index was associated with an 11.5% increase in blood loss and an increase in operative time by 12.8 minutes [7]. Similarly, Kumar et al. developed a surgical invasiveness index for patients with spinal metastatic disease [12]. Additional surgical components in the spinal metastasis invasiveness index included corpectomy, hemicorpectomy, pediculectomy, anterior column support, vertebroplasty, and use of percutaneous fixation [12]. The spinal metastasis invasiveness index predicted surgical duration (R^2^=0.28) and intraoperative blood loss (R^2^=0.18) [12]. In a comparison, the spinal metastasis invasiveness index outperformed the general spine surgical invasiveness index developed by Mirza et al. [12] Surgical invasiveness indices have also been established for adult spinal deformity patients [8, 13]. Pellise et al. included additional variables, such as implant density and team experience, and reported good discrimination for blood loss and surgical time in adult spinal deformity surgery [13]. Similarly, Neuman et al. developed a surgical invasiveness index for adult spinal deformity patients that explained 10% of the variation in operative time and 21% of the variation in estimated blood loss [8]. In a comparison between the adult spinal deformity specific index and the general spine index developed by Mirza et al., the adult deformity specific index had better performance [8]. These previous studies highlight the dependency of surgical invasiveness indices on context, such as spinal condition and patient population. Consequently, these indices cannot be directly extrapolated to the pediatric spine surgery population.

In our surgical invasiveness index, we included surgical components that are commonly used in pediatric spinal deformity surgery. Patients who received <7 posterior fusion levels without anterior fusion, pelvic instrumentation, osteotomies, or previous spinal surgery were the reference population. The mean operative time was 3.8 hours for this population, which is the base operative time. Patients with 7–12 posterior spinal fusion levels had an increase in operative time by 0.54 hours (32 minutes) and patients with >12 posterior spinal fusion levels had an increase in operative time by 1.4 hours (84 minutes). In comparison, Mirza et al. reported an average of 18.8 minutes per posterior instrumentation level [7]. In our study, patients with anterior fusion of 1–3 levels had an increase in operative time by 2.38 hours (143 minutes) and patients with anterior fusion of 4 or more levels had an increase in operative time by 2.84 hours (170 minutes). It is likely that the additional operative time associated with anterior fusion is mostly from repositioning and exposure; therefore, the operative time difference between patients who received 1–3 anterior fusion levels and patients who received 4 or more anterior fusion levels is not substantial. In the study by Mirza et al., each anterior instrumentation level was associated with 33.8 minutes increase in operative time [7]. Addition of pelvic instrumentation was associated with an increase in 0.79 hours (47 minutes). Pelvic instrumentation has been previously reported to increase operative time, surgical risk, and infection risk [14–17]. Each level of posterior column osteotomy and each level of three-column osteotomy increased operative time by 0.13 hours (8 minutes) and 0.6 hours (36 minutes), respectively. This is comparable to a study by Tetreault et al. that reported an average of 54 minutes of additional operative time for an average of 4.4 levels of posterior column osteotomies [18]. Lastly, patients that had previous spinal surgery had an increase in operative time by 0.44 hours (26 minutes). Additional surgical time may be related to scar tissue, obscured anatomical landmarks, and removal or revision of existing instrumentation. [3]

Our pediatric spine surgical invasiveness index was validated in our derivation and validation cohort using operative time and allogeneic transfusion. In our derivation cohort, the surgical invasiveness index score accounted for 16% of the variability in operative time and 14% of the variability in allogeneic transfusion. Each point in the score was associated with an increase in operative time by 0.13 hours (8 minutes). In our ROC curve analysis, the surgical invasiveness index had good discrimination for prolonged operative time and allogeneic transfusion (AUC>0.7). In our validation cohort, the surgical invasiveness index score accounted for 15% of the variability in operative time and 13% of the variability in allogeneic transfusion. Each point was associated with an increase in operative time by 0.12 hours (7 minutes). In the validation cohort, the surgical invasiveness index had good discrimination for prolonged operative time and allogeneic transfusion (AUC>0.70). We found the pediatric spine surgical invasiveness index to be predictive of operative time and allogeneic transfusion. Additionally, in our multivariable logistic regression analyses adjusted for patient factors, etiology, and use of cell salvage, the pediatric spine surgical invasiveness index was an independent risk factor for higher odds of prolonged operative time and allogeneic transfusion. Each point on the pediatric spine surgical invasiveness index was associated with a 12% increase in odds of prolonged operative time and a 9% increase in odds of allogeneic transfusion.

Surgical invasiveness indices are quantitative tools designed to measure the extent of a surgical intervention based on operative time and association with blood loss. These indices provide a standardized way to measure and compare invasiveness of procedures or surgical approaches across studies and study population. Additionally, these indices can be useful for clinical decision-making, preoperative planning, risk stratification, and outcome prediction. Cizik et al. found a score of >21 on the surgical invasiveness index developed by Mirza et al. to be associated with increased risk of surgical site infection (OR 3.15) [6]. Similarly, Hollenbeck et al. applied the surgical invasiveness index developed by Mirza et al. to adult spine patients undergoing any spinal surgery and found medium and high scores were associated with surgical site infections [19]. In adult spinal deformity patients, Arora et al. reported a surgical invasiveness index score of >36 to be associated with non-home discharge and a surgical invasiveness score of >20 to be associated with extended length of stay [20]. Hu et al. developed a thoracic spinal stenosis surgical invasiveness score that predicted transfusion rate and length of stay in patients that received open posterior spinal surgery for thoracic stenosis [21]. Passias et al. developed a cervical deformity surgical invasiveness index that included corpectomies, number of levels fused, decompression, combined approach, and radiographic parameters [9]. This cervical deformity surgical invasiveness index predicted extended length of stay, operative time, and estimated blood loss [9]. These studies in adult spine surgery patients suggest surgical invasiveness indices are useful in clinical outcomes research and can aid in risk stratification.

Understanding and using surgical invasiveness indices can have several significant clinical implications, especially in guiding surgical decision-making and resource allocation. Surgical invasiveness indices can aid clinicians in assessing the appropriateness of a surgical procedure for a patient, especially in patients that have high-risk profiles, to ensure selection of an intervention that minimizes risk. From a resource allocation standpoint, surgical invasiveness indices can aid in optimizing hospital resources and cost-efficiency. Surgical procedures that are more invasive typically have longer operative time, longer hospital stay, and more intensive postoperative care. By using invasiveness indices, hospitals can anticipate needs of each procedure and ensure appropriate allocation of resources. Surgical invasiveness indices can be a useful tool to integrate into broader risk assessments to determine a patient’s specific risk for surgery. Additionally, by understanding the invasiveness of a procedure and the associated risks, interventions such as preoperative optimization strategies, can be implemented to potentially augment the risk.

To our knowledge, this is the first surgical invasiveness index for pediatric spine surgery. Our index had good discrimination for operative time and allogeneic transfusion. There were several major limitations to this study. Our index was developed using operative time, based on the methodology described in other studies developing surgical invasiveness indices. However, unlike these studies, we validated our index using allogeneic transfusion instead of estimated blood loss. Estimated blood loss is not a variable collected by NSQIP Pediatrics. Additionally, literature suggests estimated blood loss is imprecise, inaccurate, and often underestimated, making it suboptimal for developing or validating predictive indices [22–24]. Therefore, allogeneic transfusion may be a more objective outcome to validate predictive indices. In our index, we included the most common surgical components in pediatric spinal deformity surgery. We used CPT codes to define these surgical components, which result in a lack of granularity, such as precise number of surgical levels as opposed to a range. Additionally, there could be inaccuracies associated with CPT coding that we cannot control for. Previous studies have demonstrated surgical invasiveness indices are context dependent, and, therefore, results from our study only apply to pediatric spinal deformity patients. Lastly, the focus of our study was developing and validating a surgical invasiveness index. Determining association with clinical outcomes, such as extended length of stay, adverse events, and non-home discharge, was beyond the scope of this study. Therefore, threshold values for clinical outcomes could not be established in this study. Future directions include externally validating this pediatric spine surgical invasiveness index using another study population and investigating the use of this index for risk stratification and outcome prediction.

## Supplementary Information

Below is the link to the electronic supplementary material.

Supplementary file1 (DOCX 12 KB)

## Data Availability

The National Surgical Quality Improvement Program (NSQIP) Pediatric database is available for use by all participating NSQIP centers.
