# Supplementary material for: Development and validation of a pediatric spine surgical invasiveness index
Source: Spine Deform. 2025 May 13;13(5):1367–76. doi: 10.1007/s43390-025-01106-y (PMC12401766; doi:10.1007/s43390-025-01106-y)
Supplement: Supplementary file 1 — Supplementary file1 (DOCX 12 KB) [file 43390_2025_1106_MOESM1_ESM.docx]

**SUPPLEMENTARY TABLE**

Supplementary Table 1. CPT codes

|  | CPT code(s) |
| --- | --- |
| Posterior arthrodesis for spinal deformity | 22800, 22802, 22804 |
| Anterior arthrodesis | 22551, 22552, 22556, 22558, 22585, 22808, 22810, 22812 |
| Pelvic instrumentation | 22848 |
| Posterior column osteotomies | 22210, 22212, 22214, 22216 |
| Three column osteotomies | 22206, 22207, 22208 |
